# *KRAS* Allelic Variants in Biliary Tract Cancers

**DOI:** 10.1001/jamanetworkopen.2024.9840

**Published:** 2024-05-06

**Authors:** Gordon Taylor Moffat, Zishuo Ian Hu, Funda Meric-Bernstam, Elisabeth Kathleen Kong, Dean Pavlick, Jeffrey S. Ross, Karthikeyan Murugesan, Lawrence Kwong, Anaemy Danner De Armas, Anil Korkut, Milind Javle, Jennifer J. Knox

**Affiliations:** 1 Department of Medical Oncology and Hematology, Princess Margaret Cancer Centre, University Health Network, University of Toronto, Toronto, Ontario, Canada; 2 Department of Gastrointestinal Medical Oncology, The University of Texas MD Anderson Cancer Center, Houston; 3 Department of Developmental Therapeutics, The University of Texas MD Anderson Cancer Center, Houston; 4 Department of Bioinformatics and Computational Biology, The University of Texas MD Anderson Cancer Center, Houston; 5 Foundation Medicine Inc, Cambridge, Massachusetts; 6 State University of New York Upstate Medical University, Syracuse; 7 Department of Translational Molecular Pathology, The University of Texas MD Anderson Cancer Center, Houston

## Abstract

**Importance:**

Biliary tract cancers (BTCs) contain several actionable molecular alterations, including *FGFR2*, *IDH1*, *ERBB2* (formerly *HER2*), and *KRAS*. *KRAS* allelic variants are found in 20% to 30% of BTCs, and multiple *KRAS* inhibitors are currently under clinical investigation.

**Objectives:**

To describe the genomic landscape, co–sequence variations, immunophenotype, genomic ancestry, and survival outcomes of *KRAS*-mutated BTCs and to calculate the median overall survival (mOS) for the most common allelic variants.

**Design, Setting, and Participants:**

This retrospective, multicenter, pooled cohort study obtained clinical and next-generation sequencing data from multiple databases between January 1, 2017, and December 31, 2022. These databases included Princess Margaret Cancer Centre, MD Anderson Cancer Center, Foundation Medicine, American Association for Cancer Research Project GENIE, and cBioPortal for Cancer Genomics. The cohort comprised patients with BTCs who underwent genomic testing.

**Main Outcome and Measure:**

The main outcome was mOS, defined as date of diagnosis to date of death, which was measured in months.

**Results:**

A total of 7457 patients (n = 3773 males [50.6%]; mean [SD] age, 63 [5] years) with BTCs and genomic testing were included. Of these patients, 5813 had clinical outcome data available, in whom 1000 *KRAS*-mutated BTCs were identified. *KRAS* allelic variants were highly prevalent in perihilar cholangiocarcinoma (28.6%) and extrahepatic cholangiocarcinoma (36.1%). Thirty-six *KRAS* allelic variants were identified, and the prevalence rates in descending order were G12D (41%), G12V (23%), and Q61H (8%). The variant G12D had the highest mOS of 25.1 (95% CI, 22.0-33.0) months compared with 22.8 (95% CI, 19.6-31.4) months for Q61H and 17.8 (95% CI, 16.3-23.1) months for G12V variants. The majority of *KRAS*-mutated BTCs (98.9%) were not microsatellite instability–high and had low tumor mutational burden (ranging from a median [IQR] of 1.2 (1.2-2.5) to a mean [SD] of 3.3 [1.3]). Immune profiling through RNA sequencing of *KRAS* and *NRAS–*mutated samples showed a pattern toward a more immune-inflamed microenvironment with higher M1 macrophage activation (0.16 vs 0.12; *P* = .047) and interferon-γ expression compared with wild-type tumors. The G12D variant remained the most common *KRAS* allelic variant in all patient ancestries. Patients with admixed American ancestry had the highest proportion of G12D variant (45.0%).

**Conclusions and Relevance:**

This cohort study found that *KRAS* allelic variants were relatively common and may be potentially actionable genomic alterations in patients with BTCs, especially perihilar cholangiocarcinoma and extrahepatic cholangiocarcinoma. The findings add to the growing data on genomic and immune landscapes of *KRAS* allelic variants in BTCs and are potentially of value to the planning of specific therapies for this heterogeneous patient group.

## Key Points

**Question:**

What is the value of *KRAS* allelic variants in biliary tract cancers (BTCs)?

**Findings:**

In this cohort study of 7457 patients with BTCs, *KRAS* allelic variants were common but prevalence was considerably higher among patients with perihilar and extrahepatic cholangiocarcinomas. The most common allelic variants were G12D, G12V, and Q61H, with G12D having the highest median overall survival compared with G12V and Q61H.

**Meaning:**

Findings from this study suggest that *KRAS* allelic variants within BTCs are potentially actionable genomic alterations.

## Teaser

This cohort study examines the overall survival, genomic profile, immune profile, and genomic ancestries of patients with *KRAS*-mutated gallbladder cancer or cholangiocarcinoma.

## Introduction

Biliary tract cancers (BTCs) are malignant neoplasms that emerge from the epithelium of the biliary system and are the second most common type of hepatobiliary cancer worldwide. A heterogenous group of cancers, BTCs consist of gallbladder cancer (GBC); intrahepatic cholangiocarcinoma (IHCC); and extrahepatic cholangiocarcinoma (EHCC), which includes perihilar cholangiocarcinoma (PHCC) and distal cholangiocarcinoma. The incidence and death rates of BTCs are increasing globally, and this disease remains a major health care burden.^1^ Additionally, BTCs have an overall poor outcome and a low 5-year survival rate of 5% to 15%.^2,3,4^

Surgery is the only curative treatment option for BTCs, but the majority of patients are diagnosed with metastatic or unresectable disease.^5^ For unresectable disease, the median overall survival (mOS) is between 2.5 and 4.5 months for the untreated cohort^6,7^ and approximately 1 year for the treated cohort.^8,9^ In the phase 3 TOPAZ-1 (Durvalumab or Placebo in Combination With Gemcitabine/Cisplatin in Patients With 1st Line Advanced Biliary Tract Cancer) clinical trial, the addition of the immune checkpoint inhibitor (ICI) durvalumab to gemcitabine and cisplatin vs chemotherapy alone increased mOS from 11.3 months to 12.9 months, with higher 2-year survival rates in patients with metastatic BTCs (23.6% vs 11.5%).^8^

Actionable molecular alterations have been found in approximately 30% of BTCs and include fibroblast growth factor receptor 2 (*FGFR2)* fusions, isocitrate dehydrogenase 1 (*IDH1)* sequence variations, v-raf murine sarcoma viral oncogene homologue B1 (*BRAF)* V600E sequence variations, and Erb-b2 receptor tyrosine kinase 2 (*ERBB2;* formerly *HER2*) amplifications.^10,11^ The frequency of these molecular alterations varies according to the anatomical location of the primary tumor. The *IDH1* and *FGFR2* alterations are predominantly found in IHCC, whereas *ERBB2* amplifications are often found in EHCC and GBC.^11,12,13,14^ The most common sequence variation found in BTCs includes tumor protein p53 (*TP53*), cyclin-dependent kinase inhibitor 2A and 2B (*CDKN2A* and *CDKN2B),* AT-rich interactive domain-containing protein 1A (*ARID1A),* and Kirsten rat sarcoma viral oncogene homologue (*KRAS)*.^11,15^

*KRAS* allelic variants are found in 20% to 30% of BTCs and have been associated with a more aggressive tumor phenotype and shortened survival.^16,17,18^ Multiple *KRAS* inhibitors are currently under investigation for *KRAS*-mutated tumors. In the phase 2 KRYSTAL-1 (Phase 1/2 Study of MRTX849 in Patients With Cancer Having a KRAS G12C Mutation) trial evaluating the *KRAS* G12C inhibitor adagrasib in patients with advanced solid tumors harboring a G12C variant, 12 patients with BTCs had an objective response rate (ORR) of 41.7%, a disease control rate (DCR) of 91.7%, a median progression-free survival of 8.6 months, and a mOS of 15.1 months.^19^ In addition, a post hoc exploratory analysis of the TOPAZ-1 trial in the combination immunotherapy and chemotherapy arm found that *KRAS*-mutated BTCs were more commonly found in long-term survivors who lived more than 18 months after randomization.^20^

Given the ongoing clinical development of molecular inhibitors targeting *KRAS* allelic variants and the potential synergistic property of these variants with immunotherapy, we sought to further describe the molecular and clinical features of *KRAS*-mutated BTCs. In this study, our objective was to describe the genomic landscape, co–sequence variations, immunophenotype, genomic ancestry, and survival outcomes of *KRAS*-mutated BTCs and to calculate the mOS for the most common allelic variants.

## Methods

We conducted a retrospective, multicenter, pooled cohort study of patients with BTCs using data from multiple databases. Biliary tract tumors underwent next-generation sequencing (NGS) at Princess Margaret Cancer Centre and MD Anderson Cancer Center between January 1, 2017, and December 31, 2022. Patients consented to medical record review and genomic profiling of their tumor tissue at each institution. The study was conducted in accordance with the Declaration of Helsinki.^21^ The Princess Margaret Cancer Centre and MD Anderson Cancer Center Institutional Review Boards approved the study protocols, which allowed for the retrieval of clinical, pathologic, and molecular data of patients, who signed an informed written consent or whose consent was waived. The WIRB-Copernicus Group (formerly Western Institutional Review Board) approved the use of Foundation Medicine database in this study. We followed the Strengthening the Reporting of Observational Studies in Epidemiology (STROBE) reporting guideline.

We also extracted targeted NGS and clinical data from the American Association for Cancer Research (AACR) Project Genomics Evidence Neoplasia Information Exchange (GENIE) registry, version 13.0, which encompassed 148 268 patients across 111 cancer types at 19 cancer research institutions across the world. The AACR Project GENIE is one of the largest fully public cancer genomic datasets released to date,^22^ and it contains clinical sequencing information and a limited set of clinical information, including self-reported race and ethnicity, sex, age at sequencing, cancer type sequenced using the OncoTree hierarchy, and the sample type sequenced (primary or metastatic). Additional patients were included from the following cBioPortal for Cancer Genomics databases: Memorial Sloan Kettering–IMPACT (Integrated Mutation Profiling of Actionable Cancer Targets) Clinical Sequencing Cohort,^23^ Memorial Sloan Kettering MetTropism (Metastatic Events and Tropisms),^24^ China Pan-cancer,^25^ and University of Michigan Metastatic Solid Cancers.^26^

All data were deidentified via the Health Insurance Portability and Accountability Act Safe Harbor method and incorporated into 1 database to minimize bias. Each database was unique in that each had its own set of patients and there was no overlap in database information. Patients were classified on the basis of their site of disease, such as GBC, IHCC, PHCC, or EHCC. Patients classified with EHCC primarily had distal cholangiocarcinoma. For the majority of patients included, a complete treatment history (both types and lines of therapy) was unknown, but most of the data were likely collected before the routine access to immunotherapy or ICIs to treat BTCs. Data from the Foundation Medicine database were used for genomic ancestry, immune profiling, and biomarker analyses. All quantitative variables were used as whole values to the nearest 10th decimal place and not grouped.

### Specimen Selection and Information Collection

At Princess Margaret Cancer Centre and MD Anderson Cancer Center, clinicopathologic information was retrieved from electronic medical records and included patient’s age, self-reported race and ethnicity, clinical stage at the time of diagnosis, histologic tumor subtype, tumor grade, T stage, N stage, and overall survival. The diagnosis of BTCs was confirmed by a board-certified pathologist with a subspecialty in gastrointestinal pathology. A representative formalin-fixed, paraffin-embedded (FFPE) block was chosen from each tumor specimen for performance of NGS at Princess Margaret Cancer Centre and MD Anderson Cancer Center.

### Genetic Analysis

Testing was performed on both tumor tissue and a patient-specific normal control (blood) to ensure all variant calls were somatic in nature. For the AACR Project GENIE database, all participating centers committed to providing (1) sequence variation, copy number, and gene fusion data; (2) a minimal clinical dataset of 12 data elements; and (3) a detailed accounting of the genomic regions analyzed by each assay and the specimens to which each assay was applied.

For the Princess Margaret Cancer Centre database, 2 genomic panels were used: a custom hybridization capture panel (SureSelect; Agilent) of 555 cancer-relevant genes sequenced on a NextSeq series sequencing system (Illumina), and a commercial 161-gene amplicon DNA and RNA panel (Oncomine Comprehensive Assay v3; Thermo Fisher Scientific) sequenced on the NGS platform (Ion S5 XL; Thermo Fisher Scientific).

For the MD Anderson Cancer Center database, the MD Anderson Mutation Analysis Precision Panel is a custom high-throughput NGS-based chemiluminescence immunoassay that uses targeted hybridization-based capture technology for detection of sequence variations in 610 genes (single-nucleotide variations, insertions and deletions); copy number variants in 583 genes; select gene rearrangements in 34 genes (fusions); and selected genomic immune-oncology signatures, including microsatellite instability (MSI) and tumor mutational burden (TMB) in DNA isolated from FFPE tumor tissue and cytologic specimens. Data analysis was performed in house by the MD Anderson Mutation Analysis Precision Panel bioinformatics pipeline, which relies on the dual-duplex molecular barcoding for consensus analysis to reduce sequencing artifacts and achieve greater sensitivity and positive predictive value.

For the Foundation Medicine database,^27^ comprehensive genomic profiling was performed using an adaptor-ligation or hybrid capture–based assay of coding DNA extracted from FFPE primary or metastatic colorectal cancer tumors and sequenced to high, uniform median coverage (>500 × ). Coding exons of the *KRAS* gene were selected and analyzed for base substitutions, short insertions and deletions, copy number alterations, and rearrangements.

### RNA Sequencing and Computation Analysis

RNA sequencing was performed using methods that have been previously described.^28^ The Estimation of Stromal and Immune cells in Malignant Tumours Using Expression data (ESTIMATE)^29^ algorithm was applied to assess stromal and immune cell infiltration as well as to calculate an ESTIMATE score, which is the sum of the 2 based on RNA expression data. The Cell-Type Identification By Estimating Relative Subsets of RNA Transcripts (CIBERSORT)^30^ computational method was used to assess values of 22 immune cell types with the mRNA expression data and to conduct immune cell profiling analysis. The immuno-oncology gene interaction maps package (ImogiMap, a bioinformatics tool and resources for interactions between oncogenic events and immune checkpoints associated with immunotherapy responses^31^) was used to calculate scores for epithelial to mesenchymal transition,^32^ vascularization,^33^ interferon (IFN)-γ expression score, and a T-cell inflammation gene expression signature.^34^

### Clinical Outcomes and Immunophenotype

The mOS based on *KRAS* allelic variant subtype, co–sequence variation, and primary site of tumor origin was obtained from the Princess Margaret Cancer Centre, MD Anderson Cancer Center, AACR Project GENIE, and cBioPortal for Cancer Genomics databases. Overall survival was defined as date of diagnosis to date of death, measured in months. Patients who were lost to follow-up or still alive were censored.

Immune biomarkers, including programmed death ligand 1 (PD-L1), MSI, TMB, and genome-wide loss of heterozygosity (gLOH), were derived from the Foundation Medicine database. Ancestry data were based on the 5 superpopulations from the 1000 Genomes Project: Africans, admixed Americans, East Asians, Europeans, and South Asians.^35^

### Statistical Analysis

For the primary outcome, descriptive statistics of cohort demographics, *KRAS* allelic variants, concurrent genetic aberrations, and immune biomarkers (PD-L1, TMB, MSI, and gLOH) were summarized. For survival analysis, log-rank, Wilcoxon, and Kaplan-Meier tests were conducted to compare time to death (or last seen) for each group. The designated threshold of statistical significance was *P* = .05, and no adjustment was made for multiple comparisons. All statistical analyses were conducted using SAS Studio, version 3.81 (SAS Institute Inc).

## Results

A total of 7457 patients with BTCs and genomic testing (n = 3773 males [50.6%], n = 3684 females [49.4%]; mean [SD] age, 63 [5] years) were included in the study, of whom 5813 had clinical outcome data available. Among these patients, 2499 were identified from the AACR Project GENIE, 2034 from cBioPortal for Cancer Genomics, 1644 from Foundation Medicine, 1082 from MD Anderson Cancer Center, and 198 from Princess Margaret Cancer Centre databases (Table 1). The majority of the patients had a diagnosis of cholangiocarcinoma (89%) vs GBC, and IHCC was the largest represented subtype (59%).

**Table 1.** Breakdown of Databases in Each Section of the Results

Result section	Databases
*KRAS* allelic variants, allelic frequency, and codriver variations	Princess Margaret Cancer Centre
	MD Anderson Cancer Center
	AACR Project GENIE
	cBioPortal for Cancer Genomics
Survival analysis	Princess Margaret Cancer Centre
	MD Anderson Cancer Center
	AACR Project GENIE
	cBioPortal for Cancer Genomics
Immune profiling	Foundation Medicine
	MD Anderson Cancer Center
Ancestry	Foundation Medicine

Abbreviations: AACR, American Association for Cancer Research; GENIE, Genomics Evidence Neoplasia Information Exchange.

Within the AACR Project GENIE, the patient cohort consisted of 220 males (53%) and 195 females (47%), with a mean (SD) age at diagnosis of 61 (6) years. The predominant race and ethnicity reported was White (303 [73%]), followed by Asian (25 [6%]) and Black (17 [4%]). The majority of genetic testing and DNA sequencing was completed from biopsies of the primary tumor site (228 [55%]), and 125 patients (30%) had 2 or fewer variants.

### *KRAS* Allelic Variants, Allelic Frequency, and Codriver Variations

**Figure 1.**
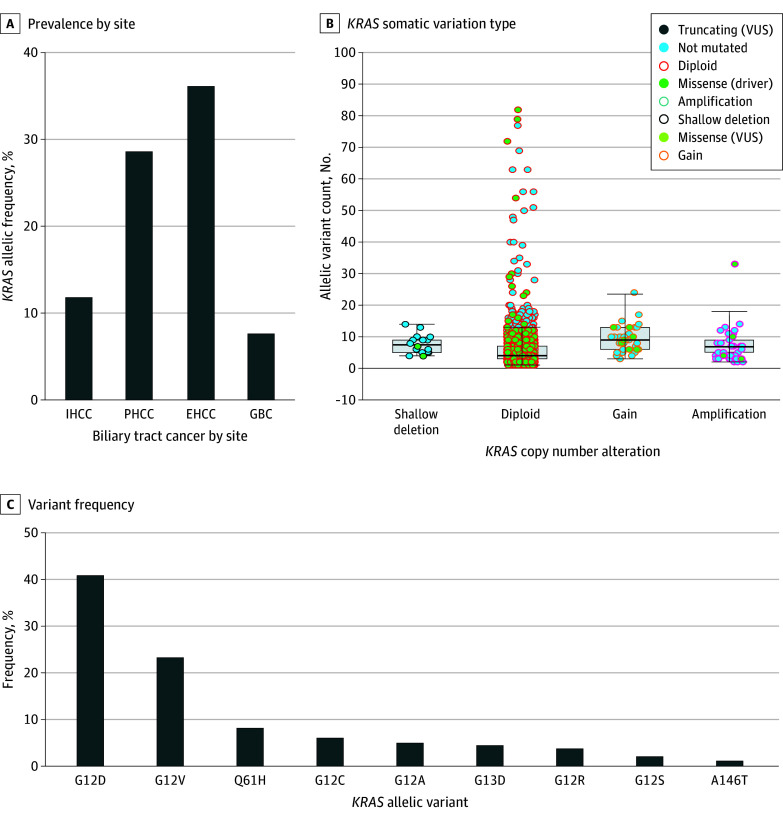
Genomic Profiling for *KRAS* Allelic Variants in Biliary Tract Cancers A, Extrahepatic cholangiocarcinoma (EHCC) and perihilar cholangiocarcinoma (PHCC) had the highest prevalence of *KRAS* allelic variants. B, *KRAS* copy number alterations and counts were obtained from The American Association for Cancer Research Project Genomics Evidence Neoplasia Information Exchange. C, G12D and G12V composed the majority of *KRAS* allelic variants in biliary tract cancer. GBC indicates gallbladder cancer; IHCC, intrahepatic cholangiocarcinoma; VUS, variant of uncertain significance.

Within the clinical cohort (n = 5813), 1000 patients had a *KRAS* allelic variant, with an overall prevalence of 17.2%. The prevalence of *KRAS* allelic variants was higher in patients with EHCC (36.1%) and PHCC (28.6%) than in those with IHCC (11.8%) and GBC (7.6%) **(**Figure 1A**)**. The most common *KRAS* allelic variant was G12D (39.5%), and the most common co–sequence variation was *TP53* (except in PHCC, which was G12V) and suppressor of mothers against decapentaplegic 4 (*SMAD4)*. The prevalence of a G12D allelic variant was 50.9% in patients with EHCC, 44.8% in patients with IHCC, and 33.3% in patients with GBC.

Based on reported data from the AACR Project GENIE, the most common *KRAS* somatic variation type was a missense variant and diploid for copy number alterations (Figure 1B). Thirty-six *KRAS* allelic variants were identified, and the prevalence rates in descending order were as follows: G12D (41%), G12V (23%), Q61H (8%), G12C (6%), G12A (5%), G13D (4%), and G12R (4%) (Figure 1C).

### Survival Analysis

**Figure 2.**
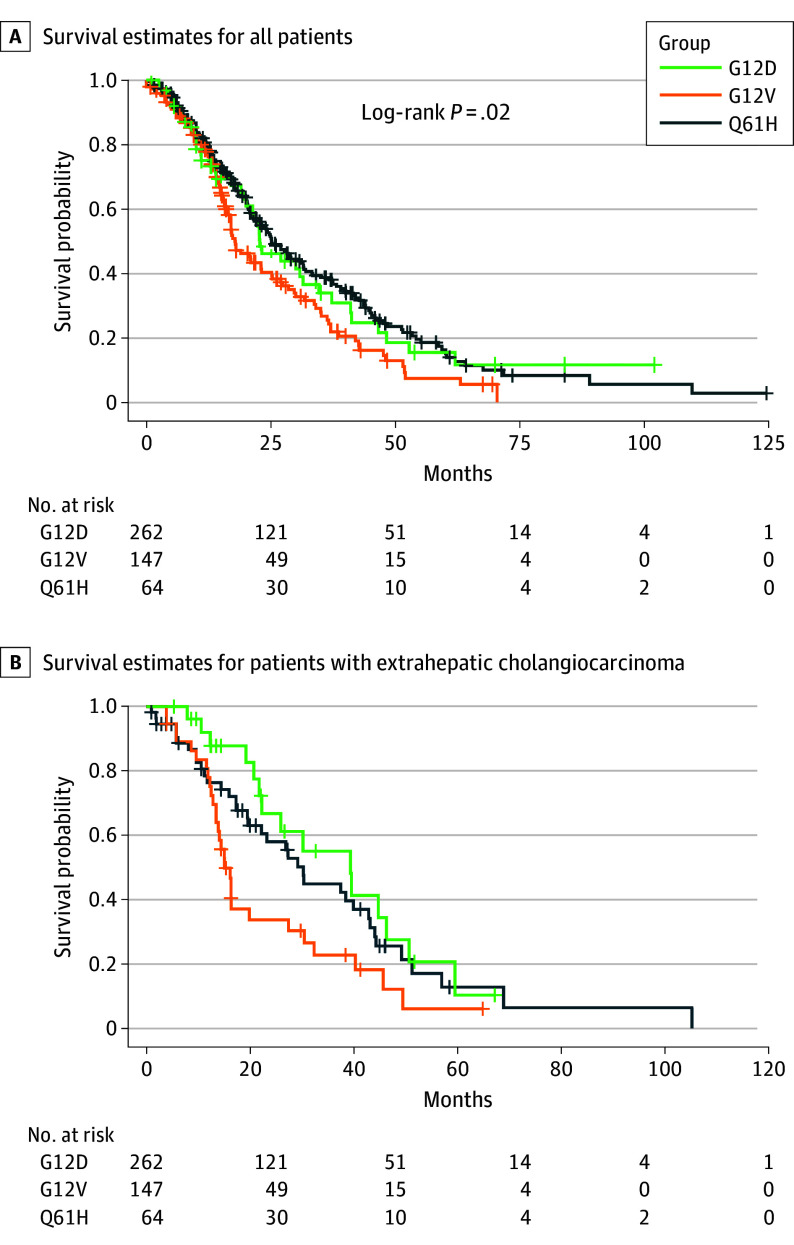
Overall Survival for *KRAS* Allelic Variants in Biliary Tract Cancers Plus signs indicate censored patients.

In the survival analysis, patients with localized and advanced disease were included. Patients with the G12D allele subtype had the highest mOS at 25.1 (95% CI, 22.0-33.0) months, followed by those with Q61H (22.8 months; 95% CI, 19.6-31.4 months) and G12V (17.8 months; 95% CI, 16.3-23.1 months) variants (*P* = .02) (Figure 2A).

In the survival analysis of *KRAS* codriver variations, there was no significant survival difference. The mOS was 20.0 (95% CI, 15.9-23.0) months for *TP53*, 21.5 (95% CI, 14.0-37.2) months for *SMAD4*, 20.4 (95% CI, 14.0-27.0) months for *CDK2NA*, and 20.9 (95% CI, 11.6-42.6) months for additional *KRAS* allelic variants (*P* = .70).

In a covariance analysis of *KRAS* allelic variants and tumor site, there was no difference in IHCC. The mOS was lower in patients with Q61H variants in PHCC and patients with G12V variants in EHCC and GBC **(**Figure 2B).

### Immune Profiling

A total of 1644 patients with BTCs and *KRAS* allelic variants were identified from the Foundation Medicine database for immune profiling and biomarker analysis (Table 2). The 3 most common *KRAS* allelic variants (G12D, G12V, and Q61H) were analyzed. The majority of *KRAS*-mutated tumors (98.9%) tested for MSI were not MSI-High. The MSI-high status was 1.5% for G12D, 1.4% for G12V, and 0.5% for Q61H. The TMB ranged from a median (IQR) of 1.2 (1.2-2.5) to a mean (SD) of 3.3 (1.3), with the higher values in patients with GBC, particularly G12V and Q61H variants, and lowest values in patients with EHCC. The highest PD-L1 positivity was among patients with GBC (24 [51%]) compared with those with IHCC (101 [37%]) and EHCC (33 [34%]). Low gLOH was common among all groups (314 of 359 [87%] in IHCC, 60 of 64 [94%] in EHCC, and 45 of 50 [90%] in GBC).

**Table 2.** Immunophenotype of *KRAS* Allelic Variants in Biliary Tract Cancers by Site

Characteristic	IHCC, No. (%)			EHCC, No. (%)			GBC, No. (%)		
	G12D	G12V	Q61H	G12D	G12V	Q61H	G12D	G12V	Q61H
Total No.	633	333	128	215	141	30	104	45	15
Sex									
Males	349 (55)	170 (51)	68 (53)	118 (55)	84 (60)	20 (67)	33 (22)	22 (49)	5 (33)
Females	284 (45)	163 (49)	60 (47)	97 (45)	57 (40)	10 (33)	71 (68)	23 (51)	10 (67)
Median (IQR) age, y	64 (25 to ≥89)	65 (23 to ≥89)	68 (28 to 85)	66 (31 to ≥89)	64 (34 to ≥89)	64.5 (31 to 86)	67 (34 to ≥89)	68 (41 to ≥89)	67 (30 to 84)
GA or tumor, No.	4.3	4.3	4.4	4.1	4.2	3.8	5.4	5	4.9
TMB									
No.	633	333	128	215	141	30	104	45	15
Median (IQR)	1.2 (0-2.5)	1.2 (0.9-2.6)	1.2 (0-2.5)	1.2 (0-2.5)	1.2 (0-2.1)	1.2 (0-2.5)	1.5 (0-3.8)	2.5 (1.2-3.8)	2.5 (1.7-4.4)
Mean (SD)	2.1 (3.0)	2.2 (2.8)	2.4 (4.1)	1.8 (3.0)	1.6 (3.7)	1.5 (1.5)	2.7 (3.2)	3.3 (5.3)	3.1 (2.0)
≥10 variants/Mb	16 (3)	7 (1)	3 (2)	3 (1)	2 (1)	0	5 (5)	2 (4)	0
≥20 variants/Mb	3 (1)	2 (1)	3 (2)	1 (1)	1 (1)	0	1 (1)	1 (2)	0
PD-L1 IHCC									
No.	158	87	28	53	34	10	29	12	6
Negativity	99 (63)	59 (67)	13 (46)	31 (58)	25 (73)	8 (80)	15 (52)	5 (42)	3 (50)
Low	46 (29)	24 (28)	12 (43)	19 (36)	9 (27)	2 (20)	9 (31)	6 (50)	1 (17)
High	13 (8)	4 (5)	3 (11)	3 (6)	0	0	5 (17)	1 (8)	2 (33)
MSI									
No.	608	320	124	204	137	28	101	45	13
MSI-High	7 (1)	2 (1)	2 (2)	1 (1)	2 (2)	0	3 (3)	1 (2)	0

Abbreviations: EHCC, extrahepatic cholangiocarcinoma; GA, genomic alterations; GBC, gallbladder cancer; IHCC, intrahepatic cholangiocarcinoma; Mb, megabase; PD-L1, programmed death 1 ligand 1; MSI, microsatellite instability; TMB, tumor mutational burden.

**Figure 3.**
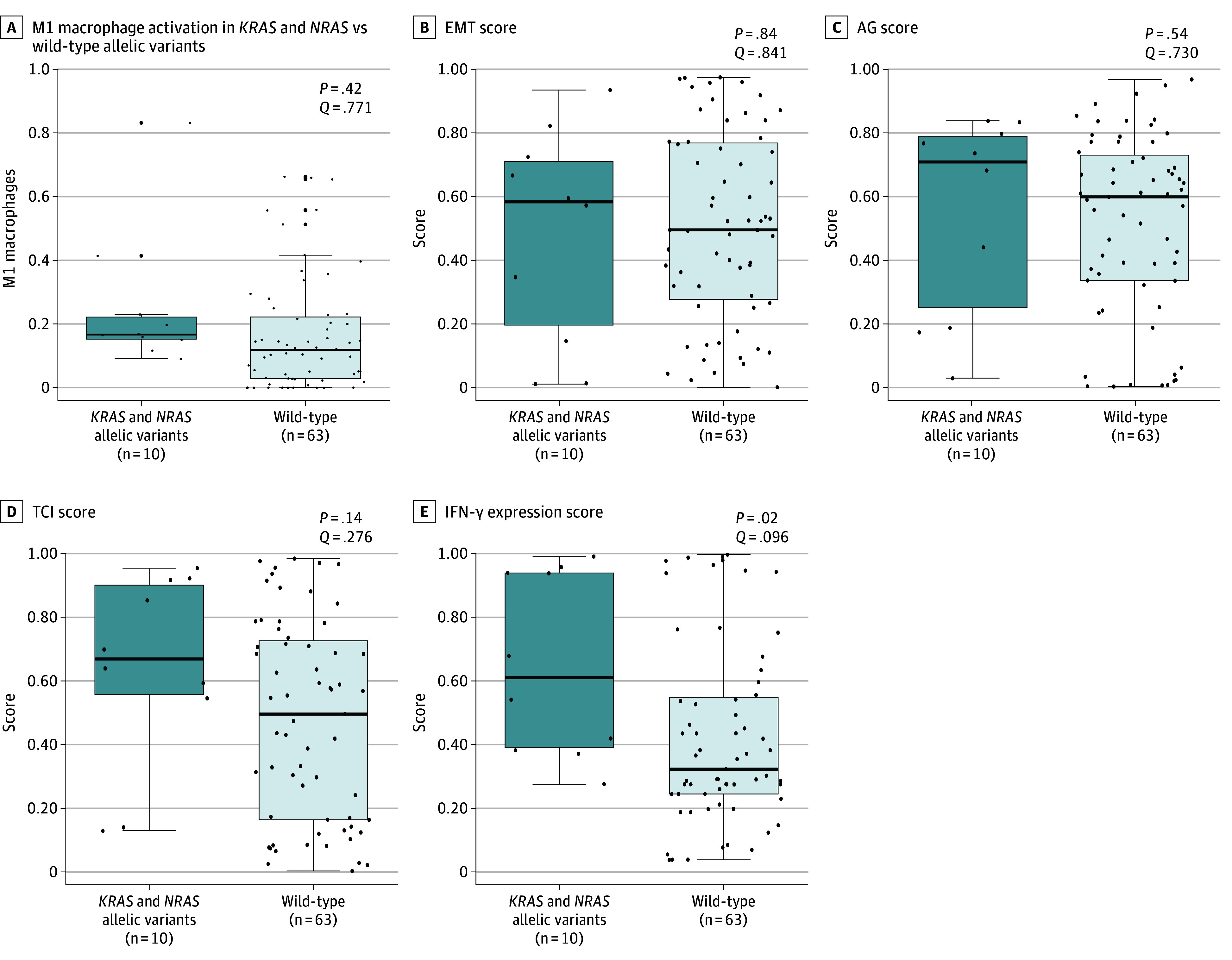
Immune Profile of *KRAS* Allelic Variants in Biliary Tract Cancers Horizontal line inside boxes represent medians; whiskers represent SDs; and dots represent individual sample score. EMT indicates epithelial to mesenchymal transition; IFN-γ, interferon gamma; TCI, T-cell inflammation.

We also assessed immune profiling through RNA sequencing of *KRAS* and *NRAS–*mutated samples obtained at MD Anderson Cancer Center and used CIBERSORT^30^ to conduct immune cell profiling analysis (Figure 3). We found that *KRAS* and *NRAS–*mutated tumors had an RNA signature that favored M1 macrophage activation over wild-type tumors (0.16 vs 0.12; *P* = .047) (Figure 3A). The distribution of immune-oncology markers in *KRAS* and *NRAS*–mutated vs wild-type samples was analyzed using the gene expression signature scores within the ImogiMap. We found that *KRAS* and *NRAS* tumors had a pattern toward a more immune-inflamed microenvironment with a higher IFN-γ expression score (Figure 3E).

### Ancestry

Using the Foundation Medicine database, we also identified the frequency of *KRAS* allelic variants based on genomic ancestry assessment. We found that the G12D variant remained the most common *KRAS* allelic variant in all patient ancestries. Patients with admixed American ancestry had the highest proportion of G12D of all the ancestries we examined (45%). Patients with East Asian and admixed American ancestries had lower proportions of the G12V variant compared with other patient ancestries (eg, 25% [East Asian] and 29% [admixed American] vs 32% [European]). Patients with East Asian and South Asian ancestries had higher proportions of the Q61H variant compared with other patient ancestries (eg, 14% [East Asian] and 11% [South Asian] vs 6% [Africans]).

## Discussion

Herein, we described the genomic and immune landscapes, clinical outcomes, and ancestries of *KRAS*-mutated BTCs. We identified 7457 patients with BTCs and 2644 patients (1000 from clinical cohort; 1644 from Foundation Medicine database) with *KRAS* allelic variants. The majority of large-sequencing studies and clinical trials for BTCs have primarily focused on identifying and targeting driver sequence variations in IHCC. Multiple *FGFR* and *IDH1* inhibitors are currently available in North America for the treatment of IHCC. Fewer therapeutic options, however, are available for patients with GBC and EHCC. In this cohort study, we found that *KRAS* allelic variants were prevalent in IHCC, EHCC, and GBCs and highly prevalent in PHCC (28.6%) and EHCC (36.1%). In reviewing genomic ancestry and immune microenvironment, we found that G12D was the most common variant in all patient ancestries but that the frequencies of other *KRAS* variants varied between ancestries. Patients with East Asian and admixed American ancestries had lower proportions of G12V compared with other patient ancestries.

The predominant *KRAS* allelic variant was G12D (39.5%), a finding consistent with previously reported results.^18,36,37^ Additionally, BTCs contained a range of *KRAS* alleles, including G12V, Q61H, G12A, G12C, and G13D. The G12V and Q61H variants had lower mOS compared with G12D, which is consistent with previous reports.^38^ Survival analysis of the *KRAS* allelic variants and their codriver variation (*TP53*, *SMAD4, CDKN2A,* or additional *KRAS* variants) was not statistically significant, suggesting that the presence of a *KRAS* oncogenic driver variation operates independent of other possible sequence variations and is associated with outcome.

Recent studies with *KRAS* inhibitors have shown promising results. In the BTC cohort of the KRYSTAL-1 trial targeting *KRAS* G12C–mutated cancers, patients treated with the *KRAS* G12C inhibitor adagrasib had an ORR of 41.7%, DCR of 91.7%, median progression-free survival of 8.6 months, and mOS of 15.1 months.^21^ However, the G12C variant was found in only approximately 6% of all *KRAS*-mutated BTCs.^39^ A number of pan-*KRAS* inhibitors are currently under development.^37,40,41^

This study highlighted the potential for combining immunotherapy with *KRAS* inhibitors in *KRAS*-mutated BTCs. The results showed that GBC had a relative higher mean and median TMB and proportion of PD-L1 high expression, which are favorable factors for ICI therapy response in *KRAS*-mutated GBC.^42^ In a 2023 study by Jeong et al,^43^ patients with *KRAS* allelic variants and PD-L1 positivity had a longer progression-free survival than patients with *KRAS* allelic variants and PD-L1 negativity. Preclinically, *KRAS* inhibitors have been associated with promotion of a proinflammatory tumor microenvironment, increased major histocompatibility complex class I protein expression, and synergies with immunotherapy to enhance antitumor activity.^44,45,46^ Moreover, *KRAS* inhibition has played a role in decreased myeloid-derived suppressor cells and increased classically activated M1-polarized macrophages, dendritic cells, cluster of differentiation (CD) 4+ cells, and CD8+ T cells, which are associated with tumor sensitivity to immune checkpoint inhibition.^45^

Preliminary data in the present study also suggest that BTCs with *RAS* sequence variation may be associated with an inflammatory tumor microenvironment. Clinically, in the KRYSTAL-7 trial, the combination of the *KRAS* G12C inhibitor adagrasib with the ICI pembrolizumab demonstrated an ORR of 49% and a DCR of 89% in patients with *KRAS* G12C–mutated advanced non–small cell lung cancer.^47^

### Limitations

This study has limitations. First, the sample was primarily composed of a North American population. We tried to overcome this limitation by including patients from the AACR Project GENIE, which includes institutions from the UK, France, Sweden, Netherlands, Spain, China, and Korea. However, a more global population may not be fully represented for interpreting the results. To our knowledge, BTCs have a growing incidence worldwide, and future studies need to include a more robust global population to support the present research. Second, each database used a different data processing and variant calling algorithm. However, given that these databases were all large cohort cancer genomic projects at major institutions within relatively the same time frame, we believe these differences were minor and did not affect the identification of a *KRAS* allelic variant nor the most common allelic variants. Third, there were fewer patients with CIBERSORT and ImogiMap analyses. Fourth, only the 3 most common *KRAS* allelic variants in this study (G12D, G12V, and Q61H) were further characterized and examined due to feasibility. Fifth, clinical data from this large cohort were limited, and the association of systemic immunotherapy with ICIs could not be assessed. Further investigation into the other *KRAS* allelic variants may unlock additional potential patterns, biomarkers, or treatment options.

## Conclusions

In this cohort study of patients with BTCs, *KRAS* allelic variants were relatively common and may be potentially actionable genomic alterations, especially in PHCC and EHCC. The most common *KRAS* allelic variant within the North American population was G12D, which has a more favorable mOS profile than G12V or Q61H. Findings from this study add to the growing data on comprehensive genomic and immune landscapes of *KRAS* allelic variants in BTCs and are potentially of value to the planning of specific therapies for this heterogeneous patient group.

## Notes

Supplement.
Data Sharing Statement
