# Supplementary material for: KRAS Allelic Variants in Biliary Tract Cancers
Source: JAMA Netw Open. 2024 May 6;7(5):e249840. doi: 10.1001/jamanetworkopen.2024.9840 (PMC11074811; doi:10.1001/jamanetworkopen.2024.9840)
Supplement: Supplement. — Data Sharing Statement [file jamanetwopen-e249840-s001.pdf]

Data Sharing Statement

Data
Data available: Yes
Data types: Deidentified participant data, Data (not involving human participants), Data dictionary
How to access data: The majority of the data that support the findings of this study are openly available in the AACR Project Genie at https://genie.cbioportal.org/study/summary?id=genie_public and the cBioPortal For Cancer Genomics at https://www.cbioportal.org/. Raw data were generated at PMCC, MDACC, and Foundation Medicine. Derived data supporting the findings of this study are available from the corresponding author on request.
When available: With publication

Supporting Documents
Document types: Statistical/analytic code, Informed consent form
How to access documents: Requests can be sent to jennifer.knox@uhn.ca
When available: With publication

Additional Information
Who can access the data: Anyone requesting the data.
Types of analyses: For any purpose.
Mechanisms of data availability: After the approval of the request.
Any additional restrictions: None.
